# Genetic polymorphisms associated with obesity in the Arab world: a systematic review

**DOI:** 10.1038/s41366-021-00867-6

**Published:** 2021-06-15

**Authors:** Salma Younes, Amal Ibrahim, Rana Al-Jurf, Hatem Zayed

**Affiliations:** Department of Biomedical Sciences, College of Health Sciences, Qatar University, Doha, Qatar

**Keywords:** Obesity, Genetics, Diseases

## Abstract

**Background:**

Obesity, one of the most common chronic health conditions worldwide, is a multifactorial disease caused by complex genetic and environmental interactions. Several association studies have revealed a considerable number of candidate loci for obesity; however, the genotype–phenotype correlations remain unclear. To date, no comprehensive systematic review has been conducted to investigate the genetic risk factors for obesity among Arabs.

**Objectives:**

This study aimed to systematically review the genetic polymorphisms that are significantly associated with obesity in Arabs.

**Methods:**

We searched four literature databases (PubMed, Science Direct, Scopus, and Google Scholar) from inception until May 2020 to obtain all reported genetic data related to obesity in Arab populations. Quality assessment and data extraction were performed individually by three investigators.

**Results:**

In total, 59 studies comprising a total of 15,488 cases and 9,760 controls were included in the systematic review. A total of 76 variants located within or near 49 genes were reported to be significantly associated with obesity. Among the 76 variants, two were described as unique to Arabs, as they have not been previously reported in other populations, and 19 were reported to be distinctively associated with obesity in Arabs but not in non-Arab populations.

**Conclusions:**

There appears to be a unique genetic and clinical susceptibility profile of obesity in Arab patients.

## Introduction

Obesity is a chronic health condition in which excessive body fat has built up to a level that may cause negative health consequences. People are considered obese when their body mass index (BMI, kg/m2) exceeds 30 kg/m^2^ [1]. Our knowledge of human obesity has progressed beyond the simple generalization that obesity is fully explained by inappropriate eating. It is now believed that obesity is a complex and multifactorial disease caused by several interactions that involve multiple factors, including genetic, metabolic, environmental, behavioral, sociodemographic, and economic [2]. Excessive fat deposition in obesity is widely considered the result of disequilibrium between energy expenditure (i.e., lack of physical activity) and energy intake (i.e., diet), resulting in excess adiposity and accumulation of lipids, primarily triglycerides, in skeletal muscle, liver, and other organs [3, 4].

The prevalence of obesity worldwide approximately tripled between 1975 and 2018 according to the World Health Organization (WHO) [1]. In 2016, over 650 million adults were obese, and 41 million children were either overweight or obese [1]. It is expected that by 2025, 17% of the world’s adults or almost 1 billion of the population will be obese, with an estimated 177 million adults predicted to become morbidly obese [5]. In the Arab world, the prevalence of obesity has drastically increased during the last three decades [6]. According to the WHO, the average prevalence of obesity in the Arab world increased from ~6.5% in 1975 to 20% in 2016 [6]. The prevalence of obesity varies significantly across the 22 Arab countries, ranging from as low as 7.8% in Comoros to as high as 37.9% in Kuwait [6]. The highest number of obesity-attributable deaths in 2016 among the 22 Arab countries was reported in Bahrain (25.69%) [6].

Arabs are a major panethnic group comprising 22 countries [7]. The ethnic composition of the Arab world has historically been altered, yielding a high degree of genetic heterogeneity [8]. It is noteworthy to mention that consanguinity rates are high in most Arab countries, with first-cousin marriage rates reaching 30% [9], this makes the architecture of the Arab genome unique with regard to their susceptibility to different diseases, including both Mendelian and complex diseases [10–20]. Given that certain ethnic groups and specific populations residing in particular geographic areas in the Arab world are more prone to obesity than others, genetic factors are believed to play a key role in predisposing certain Arab populations to obesity [21]. While genes play a fundamental role in predisposing a person to obesity, the environment can influence these genes both positively and negatively. In fact the dramatic increase in the numbers of obese people in the Arab world is believed to be largely driven by the rapid environmental changes in the Arab world that have occurred in the last two decades, which have brought significant prosperity, lifestyle changes, and urbanization to the Arab world [22]. These factors have created an “obesogenic environment” in Arab countries, and thus, genetic and environmental factors are believed to have a balance that variably intertwines in the development of obesity [22].

With the rising prevalence of obesity in the Arab world, there has been a growing interest in understanding the genetic architecture that renders Arabs susceptible to obesity. Over the past decade, researchers have started to sequence Arab genomes through national projects in hopes of defining disease-associated genetic polymorphisms for gene disorders in Arabs and to establish meaningful genotype–phenotype correlations. Starting with Saudi Arabia [23], followed by Qatar [24], and currently the United Arab Emirates [25], these countries are establishing their 1000 Genomes Projects. These projects are crucial for mapping potential obesity-associated genetic variants among Arabs to help characterize Arab patients suffering from obesity, which may ultimately improve premarital genetic counseling, health outcomes and quality of life in obese Arab patients.

Obesity etiology is known to be multifactorial, involving a complex interplay between genes and environment [26]. While a genetic basis for obesity does exist, defining the exact genetic contribution has proven to be a difficult task [26]. The genetic heritability of obesity is estimated to account for 30–50% of the age- and gender-adjusted phenotypic variances [26]. Approximately 31–90% of interindividual body weight variability is attributed to genetic factors [27, 28]. As many as 200 genetic variants in various genome-wide association studies (GWASs) have been associated with obesity in many different populations, predominantly Europeans. Nevertheless, only about 3% of the heritability can be explained by variants currently known to be associate with BMI [29].

According to the genetic criteria, obesity is classified into: (i) monogenic, when a mutated gene is responsible for the phenotype; (ii) syndromic, when a set of specific symptoms are present, and a small group of genes is involved; and (iii) polygenic, also referred to as “common” obesity, which accounts for 95% of obese cases, with many genes adding up to provide a further risk to the individual [30].

Given the complex nature of obesity and the fact that it does not follow a typical Mendelian transmission pattern, it is believed that several susceptibility genes with low or moderate effects play a role in predisposition to the disease [31, 32]. There is firm evidence that genes influencing energy homeostasis and thermogenesis, adipogenesis, leptin-insulin signaling transduction, and hormonal signaling peptides play a role in the development of obesity [33–37]. Several potential obesity candidate genes and variants have been documented, including *LEP* and *LEPR*, which have been mainly implicated in monogenic obesity, as well as *FTO*, *APOE, PPARG*, and *PPARA* which have been mainly implicated in polygenic/common obesity [31, 32]. However, the genotype–phenotype correlations remain unclear. In addition, there has been relatively little attention devoted to comprehensively investigating genetic polymorphisms associated with obesity in Arab populations. Therefore, in this study, we aimed to systematically review the current evidence on genetic variants associated with monogenic and polygenic obesity in the Arab world.

## Methods

To ensure the rigor of the current systematic review, it was designed and implemented based on the Preferred Reporting Items for Systematic Reviews and Meta-analyses (PRISMA) guidelines [38] (Table S1). The described search method and selection strategy were used to identify studies investigating the effect of obesity-associated genetic variants, including single nucleotide polymorphisms (SNPs), variable number tandem repeats (VNTRs), deletions, insertions, and copy-number variants, on obesity risk in Arab patients residing in any of the 22 Arab countries.

### Search strategy

We used four literature databases (PubMed, Scopus, Science Direct, and Google Scholar) to retrieve all studies related to obesity genetics in Arab populations up to May 2020. In addition, cross-referencing from the bibliographies of all retrieved articles (citation tracking) was performed. We utilized several Boolean operators and search strings for the different electronic database searches (Table S2).

To gain a better understanding of the ethnic distribution of the captured variants and whether they are distinctive to Arab populations (i.e., if they circulate only among Arabs but not in other ethnic groups), we individually searched the captured variants in the following databases: Leiden Open Variation Database (LOVD) (http://www.lovd.nl/), ClinVar (https://www.ncbi.nlm.nih.gov/clinvar/), dbSNP (https://www.ncbi.nlm.nih.gov/snp/), Human Genome Mutation Database (HGMD) (http://www.hgmd.cf.ac.uk/ac/index.php), Exome Variant Server (EVS) (http://evs.gs.washington.edu/EVS/), PubMed, and Google Scholar.

### Study selection

**Fig. 1:**
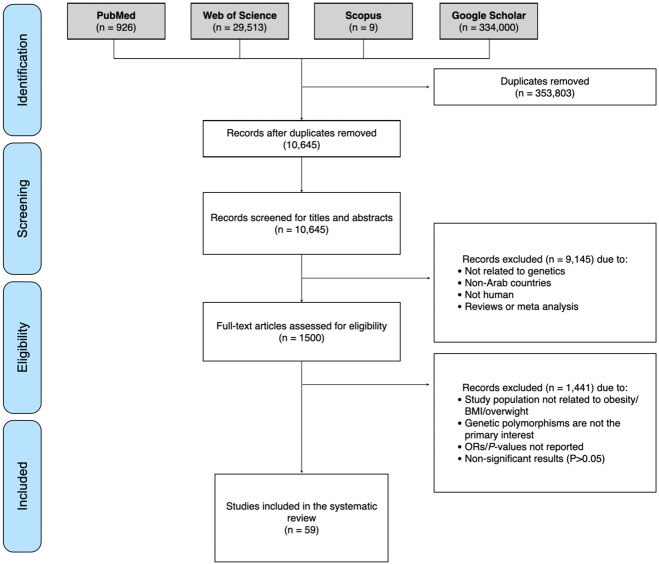
PRISMA flow diagram of the included studies. A total of 1500 full-text articles were assessed for eligibility, of which 59 studies met our inclusion criteria and were included in the systematic review.

The inclusion and exclusion criteria were developed using a PECO(T) [participants, exposure, comparator, outcome(s), and type of study] structure. However, as we are looking for observational studies, we only considered participants, exposure and the outcome of interest. For inclusion, studies had to meet all the following criteria: (1) Population: Arabs residing in Arab countries. (2) Exposure: genetic polymorphisms significantly associated with obesity (*P* < 0.05). (3) Outcome: obesity (BMI ≥ 30 kg/m^2^). We included all studies reporting obesity indices or anthropometric measures, such as BMI, fat mass, waist circumference (WC), hip circumference (HC), and waist-hip ratio (WHC). (4) Type of study: no study type limits were applied. Articles published in peer-reviewed journals were included. We excluded duplicate publications, studies on animal subjects, studies conducted on non-Arabs, case series, review articles, and articles not in the English language. The PRISMA flow chart for study selection is shown in Fig. 1.

Three scientists (SY, AI, and R.A.-J) worked independently in identifying, screening, and performing the quality control of the extracted data from the four literature databases. All citations were exported to Endnote X9, and duplicate citations were removed. Articles were screened in two stages: [1] the first stage involved performing the initial screening of the titles and abstracts, and assessing relevance for the scope of the systematic review according to our inclusion/exclusion criteria, [2] the second stage involved retrieval of the full text of each potentially relevant study and screening for content to decide on its inclusion. Articles for which full-text articles could not be retrieved or only abstracts were available were reviewed for content and were included if they met the inclusion criteria. Points of discrepancies were resolved through discussion between SY, AI, and R.A.-J, and additional points of debate were further resolved with HZ until a consensus was reached.

### Data extraction

The following information was extracted and recorded: genes associated with obesity, chromosomal location, gene function, significantly associated variant (*P* < 0.05), SNP ID, country of origin, clinical phenotype reported in the study, cases (*n*), controls (*n*), gender and age (if mentioned), the number of individuals screened for gene variants vs number of obese individuals carrying variants, associated allele/genotype, methods (including baseline characteristics and genotyping techniques) and statistical data [crude odds ratios (cOR) and adjusted (aOR), 95% confidence interval (CI), *P* value]. Finally, the ethnic distribution of each variant was recorded. A thorough search was conducted in public databases (ClinVar and dbSNP) for any missing information.

### Quality assessment

Quality assessment was performed using the assessment tools of the National Heart, Lung, and Blood Institute of the National Institutes of Health (NIH): [1] the NIH Quality Assessment tool for Observational Cohort and Cross-Sectional Studies; and [2] the NIH Quality Assessment tool for Case-Control Studies [39]. The NIH tools categorizes studies as either good, fair, or poor. Table S4 summarizes the quality assessments of the 59 studies that were included in this systematic review.

## Results

**Fig. 2:**
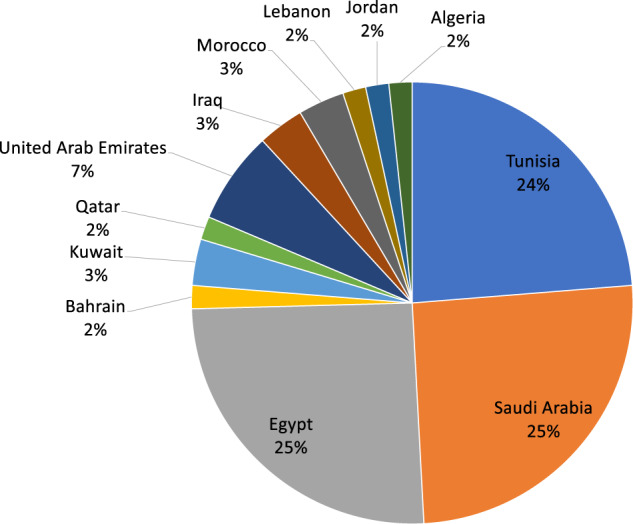
Classification of the included studies according to country. Most of the studies were conducted in Saudi Arabia (25%) and Egypt (25%), followed by Tunisia (24%). Fewer studies were conducted in the United Arab Emirates (7%), Kuwait (3%), Iraq (3%), Morocco (3%), Bahrain (2%), Qatar (2%), Lebanon (2%), Jordan (2%), and Algeria (2%). No genetic data were captured from the remaining 10 Arab countries (Yemen, Oman, The Comoros Islands, Djibouti, Mauritania. Somalia, Sudan, Libya, Syria, or Palestine). The number of captured variants was 76 located within or near 49 genes.

A total of 1500 full-text articles were assessed for eligibility, among which 59 studies met our inclusion criteria and were included in the systematic review (Fig. 1). Most of the studies were case-control studies (*n* = 49). The remaining were cross-sectional (*n* = 9) and cohort GWA analysis (*n* = 1). The studies included in the systematic review comprised a total of 15,488 cases and 9760 controls. The studies captured Arabs from 12 Arab countries, and most of the studies were conducted in Saudi Arabia (*n* = 15) and Egypt (*n* = 15), followed by Tunisia (*n* = 14). Fewer studies were conducted in the United Arab Emirates (*n* = 4), Kuwait (*n* = 2), Iraq (*n* = 2), Morocco (*n* = 2), Bahrain (*n* = 1), Qatar (*n* = 1), Lebanon (*n* = 1), Jordan (*n* = 1), and Algeria (*n* = 1) (Fig. 2). No genetic data were captured from the remaining ten Arab countries (Yemen, Oman, The Comoros Islands, Djibouti, Mauritania. Somalia, Sudan, Libya, Syria or Palestine). The number of captured variants comprised 76 located within or near 49 genes (Table 1, Table S3, and Fig. 2). Data are summarized in Table 1 and Table S3. Among the 49 genes included in this systematic review, *FTO* was the most frequently studied gene in Arab patients with obesity (reported in 14 different articles) (Table 1, Table S3). In this systematic review, variants in the *FTO* gene were captured from six Arab countries (Saudi Arabia, Tunisia, Egypt, Iraq, Kuwait, and United Arab Emirates) (Table 1, Table S3). Other commonly studied genes were *LEP* (*n* = 7), *ADIPOQ* (*n* = 4), and LEPR (*n* = 3). All remaining genes were reported only once or twice (Table 1).

**Table 1:** Genetic polymorphisms associated with obesity retrieved from the 59 included studies.

Gene	Variant	SNP ID	Country	Cases (*n*)	Controls (*n*)	Clinical phenotype	OR	95% CI	*P* value	Reference
*ABCA1*	G > A	rs2230806	Egypt	128	128	Obesity	2.75	1.19–6.36	0.042	[69]
*ACE*	287-bp Alu ins/del in intron 16	–	Tunisia	259	302	Obesity	NR	NR	NR	[70]
*ACE*	287-bp Alu ins/del in intron 16	–	Egypt	70	72	Obesity	3.45	1.26–9.48	0.005	[71]
*ADIPOQ*	+2019delA	–	Tunisia	160	169	Obesity	NR	NR	NR	[72]
*ADIPOQ*	c.11374 C > G	–	Tunisia	160	169	Obesity	NR	NR	NR	[72]
*ADIPOQ*	c.164 C > T	–	Jordan	389	–	Obesity	NR	NR	0.000	[73]
*ADIPOQ*	G > T	rs1501299	Egypt	100	97	Obesity	4.23	2.8–6.4	0.000	[74]
*ADIPOQ*	G > T	rs1501299	Jordan	389	–	Obesity	NR	NR	0.000	[73]
*ADIPOQ*	G > T	rs1501299	Tunisia	160	169	Obesity	0.64	0.409–0.917	0.039	[72]
*ADIPOQ*	G > T	rs1501299	Tunisia	400	721	Obesity	0.603	0.467–0.779	0.001	[75]
*ADIPOQ*	−11391 G/A	rs17300539	Tunisia	160	169	Obesity	1.68	1.040–3.110	0.044	[72]
*ADIPOQ*	c.639 C > T	rs3821799	Tunisia	160	169	Obesity	1.85	1.110–3.100	0.018	[72]
*ADIPOQ*	c.4552 C > T	rs822393	Tunisia	400	721	Obesity	1.52	1.230–1.994	0.001	[75]
*ADRB2*	Arg16Gly	rs1042713	Saudi Arabia	214	115	Obesity	3.38	1.456–7.822	0.0001	[76]
*ADRB3*	Trp64Arg	rs4994	Saudi Arabia	214	115	Obesity	7	2–24	0.0004	[77]
*AGT*	M235T	rs699	Tunisia	259	302	Obesity	0.29	0.19–0.44	0.001	[70]
*APLN*	–	rs3115757	Egypt	112	39	Obesity	12.09	1.39–104.85	0.024	[78]
*APOA2*	c.−492T > C	–	Egypt	196	107	Obesity	NR	NR	0.001	[79]
*APOA2*	c.−265T > C	rs5082	Egypt	240	260	Obesity	NR	NR	0.001	[80]
*APOA5*	c.56 C > G	rs3135506	Morocco	164	295	Obesity	3.16	1.52–6.62	0.002	[81]
*APOA5*	c.−1131T > C	rs662799	Morocco	164	295	Obesity	4.13	1.86–9.03	0.0001	[81]
*ARAP1*	G > T	rs1552224*	Saudi Arabia	1800	423	Obesity	1.5	1.0–2.3	0.04	[82]
*ASAH1*	C > T	rs17126232	United Arab Emirates	80	–	Obesity	NR	NR	NR	[83]
*BDNF*	T > A	rs10767664	Saudi Arabia	204	NR	Obesity	1.923	1.32–2.81	0.00072	[84]
*CD36*	G > A	rs1761667	Algeria	83	82	Obesity	1.63	1.04–2.55	0.041	[85]
*ENPP1*	K121Q	rs1044498	Morocco	503	412	Obesity	1.91	1.27–2.85	0.002	[86]
*EXT2*	–	rs3740878*	Egypt	37	37	Obesity	6	2.0–17.6	0.001	[87]
*FTO*	G > T	rs17817449	Egypt	37	37	Obesity	2.4	1.0–5.5	0.036	[87]
*FTO*	G > T	rs17817449	Saudi Arabia	106	106	Obesity	1.75	1.02–3.03	0.043	[88]
*FTO*	G > T	rs3751812	United Arab Emirates	318	392	Obesity	1.47	1.09–1.99	0.011	[89]
*FTO*	G > T	rs3751812	Saudi Arabia	204	NR	obesity	1.523	1.08–2.15	0.016	[84]
*FTO*	C > A	rs11642841	Saudi Arabia	1800	423	Obesity	1.5	1.1–2.1	0.01	[82]
*FTO*	T > C	rs1421085	United Arab Emirates	915	–	Obesity	NR	NR	0.0041	[90]
*FTO*	T > C	rs1421085	Saudi Arabia	136	104	Obesity	2.5	1.13–5.55	0.023	[91]
*FTO*	T > A	rs9939609	Egypt	110	122	Obesity	1.89	1.29–2.78	0.001	[32]
*FTO*	T > A	rs9939609	Saudi Arabia	20	166	Obesity	NR	NR		[92]
*FTO*	T > A	rs9939609	Kuwait	674	214	Obesity	1.47	1.01–2.12	0.041	[93]
*FTO*	T > A	rs9939609	United Arab Emirates	315	98	Obesity	NR	NR	0.027	[90]
*FTO*	T > A	rs9939609	Iraq	120	50	Obesity	NR	NR	NR	[94]
*FTO*	T > A	rs9939609	Iraq	120	60	Obesity	NR	NR	0.031	[95]
*FTO*	T > A	rs9939609	Saudi Arabia	136	104	Obesity	2.97	1.30–6.79	0.009	[91]
*FTO*	T > A	rs9939609	Tunisia	494	334	Obesity	1.7	1.29–2.25	0.001	[96]
*GC*	GT	rs7041*	Bahrain	406	–	Obesity	NR	NR	0.012	[97]
*GCKR*	T > C	rs780094	Egypt	37	37	Obesity	3.6	1.6–7.9	0.001	[87]
*GNB3*	c.825 C > T	rs5443	Saudi Arabia	106	106	Obesity	6.68	3.26–13.69	0.0001	[88]
*GNPDA2*	A > G	rs10938397	Qatar	614	190	Obesity	2.4	1.27–4.56	0.0266	[98]
*IGF2BP2*	G > T	rs4402960	Tunisia	256	90	Obesity and T2D	2.06	1.40–3.03	0.0001	[99]
*IL-6*	G > T	rs1554606*	Saudi Arabia	204	–	Obesity	1.55	1.08–2.22	0.024	[100]
*IL-6*	c.174 G/C	rs1800795*	Egypt	85	64	Obesity	NR	NR	0.000	[101]
*LEP*	c.104 T > G**	–	Egypt	80	–	Obesity	NR	NR	NR	[36]
*LEP*	c.34delC**	–	Egypt	80	–	Obesity	NR	NR	NR	[36]
*LEP*	c.313 C > T	rs104894023	Egypt	80	–	Obesity	NR	NR	NR	[36]
*LEP*	3’ UTR A/C	rs11761556*	Tunisia	160	169	Obesity	2.63	1.13–6.11	0.025	[102]
*LEP*	G/A	rs1349419	Tunisia	27	6	Obesity	NR	NR	0.039	[65]
*LEP*	G > A	rs2167270	Tunisia	27	6	Obesity	NR	NR	0.039	[65]
*LEP*	c.2548 G > A	rs7799039*	Tunisia	160	169	Obesity	1.87	1.106–2.78	0.028	[102]
*LEP*	c.2548 G > A	rs7799039*	Tunisia	400	721	Obesity	2.36	1.75–3.18	0.001	[75]
*LEP*	c.2548 G > A	rs7799039*	Tunisia	160	169	Obesity and MetS	3.405	1.78–6.48	0.001	[103]
*LEP*	c.2548 G > A	rs7799039*	Tunisia	229	251	Obesity	NR	NR	0.001	[64]
*LEP*	Tetranucleotide repeat (TTTC)n		Egypt	120	83	Obesity and MS	14.6	5.5–38.6	0.000	[66]
*LEPR*	p.Lys109Arg	rs1137100	Tunisia	160	169	Obesity	0.399	0.179–0.892	0.025	[102]
*LEPR*	p.Gln223Arg	rs1137101	Tunisia	160	169	Obesity	1.41	1.035–1.85	0.045	[102]
*LEPR*	p.Gln223Arg	rs1137101	Egypt	110	122	Obesity	2.21	1.30–3.74	0.004	[32]
*LEPR*	c.3057 G > A	rs62589000	Tunisia	393	317	Obesity	2.73	1.03–7.21	0.042	[31]
*LOC284260*, *RIT2*	G/A	rs7239883	United Arab Emirates	915	–	Obesity	NR	NR	0.0075	[104]
*LPL*	C > T	rs285*	Egypt	120	83	Obesity	6.6	1.9–22.9	0.000	[105]
*MC4R*	T > C	rs17782313	United Arab Emirates	318	392	Obesity	1.35	1.00–1.81	0.048	[89]
*MC4R*	T > C	rs17782313	Saudi Arabia	136	104	Obesity	1.72	1.02–2.89	0.038	[91]
*MC4R*	C > A	rs571312	United Arab Emirates	318	392	Obesity	1.39	1.03–1.87	0.03	[89]
*MMP-1*	−519 A/G	rs1144393*	Tunisia	168	202	Obesity	1.61	1.07–2.44	0.02	[106]
*MMP-12*	c.82 A > G	rs2276109*	Tunisia	168	202	Obesity	2.39	1.36–4.2	0.002	[106]
*MMP-3*	Lys45Glu (A > G)	rs679620	Tunisia	168	202	Obesity	4.93	1.84–13.32	0.002	[106]
*MMP-7*	−181 A/G	rs11568818*	Tunisia	168	202	Obesity	3.68	1.40–6.71	0.01	[106]
*MTHFD1*	c.1958G > A	rs2236225*	Egypt	37	37	Obesity	4.6	0.9–23.6	0.050	[87]
*MTHFR*	c.677 C > T	rs1801133	Egypt	51	30	Obesity	13.5	2.82–64.67	0.001	[107]
*NOS3*	c.894 G > T	rs1799983	Tunisia	183	211	Obesity	2.62	0.99–6.92	0.04	[108]
*NOS3*	4a/b*		Tunisia	183	211	Obesity	1.72	1.16–2.56	0.004	[108]
*PPARG*	C > G	rs1801282	Egypt	37	37	Obesity	3.4	1.6–7.2	0.001	[87]
*PPARG*	C > G	rs1801282	Tunisia	262	83	Obesity	3.4	1.6–7.2	0.001	[109]
*PRDM16*	–	rs2651899*	Saudi Arabia	89	84	Obesity	44.6	11.5984–172.0157	0.0001	[110]
*PROX1*	–	rs1704198	United Arab Emirates	80	–	Obesity	NR	NR	NR	[83]
*PTGS1*	–	rs5788*	Egypt	37	37	Obesity	3.3	1.4–7.9	0.006	[87]
*RETN*	−420 C > G	rs1862513*	Egypt	145	155	Obesity	3.06	1.49–6.26	0.014	[111]
*RETN*	−420 C > G	rs1862513*	Tunisia	400	721	Obesity	1.48	1.09–2	0.011	[75]
*RETN*	−420 C > G	rs1862513*	Tunisia	160	169	Obesity and MetS	2.22	1.39–3.56	0.001	[112]
*RETN*	c.+299 G > A	rs3745367	Egypt	145	155	Obesity	3.53	1.65–7.52	0.005	[111]
*STAT4*	G > T	rs7574865*	Egypt	37	37	Obesity and diabetes	4	1.7–9.5	0.001	[87]
*TCF7L2*	C > T	rs7903146	Saudi Arabia	1800	423	Obesity	1.4	1.0–1.8	0.03	[82]
*TCF7L2*	C > T	rs7903146	Lebanon	308	–	Obesity	NR	NR	0.001	[113]
*TCN2*	c.67 A > G	rs9606756*	Kuwait	1260	1272	T1D	NR	NR	0.0132	[114]
*TFAP2B*	A > G	rs987237	Qatar	614	190	Obesity	0.79	0.54–1.15	0.0014	[98]
*TMEM18*	A > C	rs12463617	United Arab Emirates	318	392	Obesity	0.7	0.51–0.95	0.023	[89]
*TP53*	C > G	rs1042522*	Saudi Arabia	136	122	Obesity	2.169	1.086–4.334	0.02716	[115]
*UCP1*	A > G	rs1800592	Saudi Arabia	337	155	Moderate and extreme obesity	1.52	1.10–2.08	0.009	[116]
*UCP1*	–	rs3811791	Saudi Arabia	337	155	Moderate obesity	2.89	1.33–6.25	0. 007	[116]
*UCP2*	45-bp ins/del	–	Saudi Arabia	86	65	Severe obesity	0.18	0.07–0.44	0.0004	[117]
*UCP2*	45-bp ins/del	–	Saudi Arabia	84	29	Obesity and T2D	0.18	0.07–0.44	0.0004	[118]
*USP37*	–	rs492400	United Arab Emirates	915	–	Obesity	NR	NR	0.0096	[104]
*VDR*	Bsm-I	rs1544410	Saudi Arabia	402	489	Obesity	NR	NR	0.028	[119]
*VDR*	Bsm-I	rs1544410	Saudi Arabia	200	100	Obesity	NR	NR	0.042	[120]
*VDR*	TaqI	rs731236	Saudi Arabia	402	489	Obesity	NR	NR	0.009	[119]
*VDR*	TaqI	rs731236	Saudi Arabia	200	100	Obesity	NR	NR	0.021	[120]
*VDR*	TaqI	rs731236	Egypt	50	60	Obesity	2.33	1.34–4.1	0.003	[121]

*CI* confidence interval, *OR* odds ratio, *NR* not reported

*Unique genotype–phenotype correlation.

**Unique variant.

According to the number of variants reported per gene, most variants were captured in *LEP*, with eight obesity-associated variants reported among Arabs (rs11761556, rs7799039, c.104 T > G, c.34delC, rs104894023, rs1349419, rs2167270, and tetranucleotide repeat (TTTC) *n*) from 7 studies that were conducted in two Arab countries (Tunisia and Egypt) (Table 1, Table S3). The remaining variants were primarily captured in *ADIPOQ* (7 variants), *FTO* (5 variants), and *LEPR* (3 variants). The remaining genes had one or two variants (Table 1, Table S3). Among all 76 variants captured in this study, *FTO* rs9939609 was the most commonly studied variant, reported in eight studies conducted in six different countries (Egypt, Saudi Arabia, Tunisia, Iraq, Kuwait, and United Arab Emirates) in association with obesity (Table 1, Table S3).

**Fig. 3:**
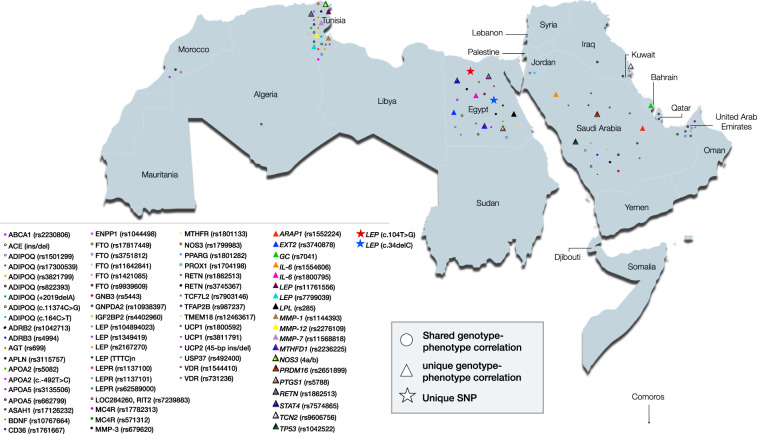
Geographic distribution of obesity-associated genetic polymorphisms in the Arab world. A total of 76 variants were reported to be significantly associated with obesity. Among the 76 variants, 55 exhibited shared genotype–phenotype correlation, 19 showed unique genotype–phenotype correlation, and two were described as unique to Arabs, as they were not previously reported in other ethnicities.

Among the 76 variants in 49 different genes, two variants were unique to Arabs (*LEP*: c.104 T > G, c.34delC) (i.e., reported in Arabs and not reported in any other ethnic groups) (Fig. 3). The remaining 74 variants were classified into two different categories based on their genotype–phenotype correlation. The first category included variants with unique genotype–phenotype correlations in Arabs; i.e., they are distinctively associated with obesity among Arabs but have been reported in association with clinical conditions other than obesity among different ethnicities (19 variants) (Fig. 3). The second category included variants that were reported in association with obesity in Arabs as well as other non-Arab ethnic populations and were thus considered common variants, i.e., shared with other ethnicities (55 variants) (Fig. 3). Data on the ethnic distribution of the captured variants is summarized in Table S3.

Statistical data from the 59 individual studies included in this systematic review are shown in Table S3. Overall, 57 studies reported cORs, which ranged from 0.18 to 44.60, with a median of 2.39 (interquartile range 1.62–3.57), and 49 studies reported aORs that ranged from 0.07 to 13.76, with a median of 1.65 (interquartile range 1.29–2.18) (Table S3). Among the 57 studies which reported cORs, *PRDM16:* rs2651899 conferred the strongest association with obesity (cOR = 44.60, 95% CI: 11.60–172.02, *P* = 0.0001). Among the 49 studies, which adjusted for confounders, *MTHFR:* rs1801133 conferred the strongest adjusted association (aOR = 13.5, 95% CI: 2.82–64.67, *P* = 0.001).

## Discussion

To our knowledge, this is the first systemic review to comprehensively summarize all genetic polymorphisms associated with the risk of developing obesity in Arab patients. In this systematic review, we captured 76 genetic variants from 59 eligible studies, comprising 15,488 cases and 9760 controls. We identified 76 variants in 49 genes (Table 1, Table S3). Among the 49 genes that have been reported, the *FTO* gene, reported in 14 different articles, was the most frequently studied gene among Arab patients with obesity. The *FTO* gene is one of the most common genes that has been studied around the world in association with obesity [40]. *FTO* is highly expressed in the hypothalamus and functions as a regulator of appetite and energy expenditure [41–44] (Table S3). In this study, five captured variants were located in the *FTO* gene, all of which are intronic variants (rs11642841, rs1421085, rs17817449, rs3751812, rs9939609), reported in six Arab countries (Saudi Arabia, Tunisia, Egypt, Iraq, Kuwait, and United Arab Emirates) (Table 1, Table S3). The variant *FTO*: rs9939609 was the most commonly studied variant among all 76 variants captured in this study (Table 1), reported eight times in six different countries (Egypt, Saudi Arabia, Tunisia, Iraq, Kuwait, and United Arab Emirates) in association with obesity (Table 1, Table S3). *FTO*; rs9939609 has been previously reported in several other Non-Arab ethnicities in association with energy intake, eating habits, and susceptibility to obesity [45–51].

According to the number of variants reported per gene, most were captured in *LEP*, with eight obesity-associated variants reported among Arabs (rs11761556, rs7799039, c.104 T > G, c.34delC, rs104894023, rs1349419, rs2167270, and tetranucleotide repeat (TTTC) *n*) from seven studies conducted in two Arab countries (Tunisia and Egypt) (Table 1). The remaining variants were primarily captured in *ADIPOQ* (7 variants), *FTO* (5 variants), and *LEPR* (3 variants). The remaining genes had one or two variants each (Table 1).

Among the 76 variants captured in this systematic review, two were unique to Arabs, as they were not previously reported in any other ethnic groups and were identified in the original study as novel, both of which are located in *LEP* (c.104 T > G, c.34delC) (Fig. 3).

### Variants unique to Arab countries

Two variants were found to be unique to Arabs, both of which are located in *LEP* (c.104 T > G and c.34delC) [36] (Table 1). These two variants were identified as novel homozygous variants and were not previously reported in any database. The missense variant c.104 T > G was identified in a 7‐month‐old boy who presented with excessive weight gain. This variant resulted in an amino acid substitution from isoleucine to serine at position 35 (p. Ile35Ser) and was predicted to likely be pathogenic [36] (Table 1, Table S3). The variant c.34 delC is a deletion of a cytosine nucleotide at position 34, causing a frameshift in the protein and changing the leucine at position 12 of the protein [36] (Table 1, Table S3). Functional analysis revealed that these two variants are likely to be disease‐causing [36]. The *LEP* gene has globally been reported to contribute to the pathogenesis of obesity [52]. *LEP* regulates the leptin-melanocortin signaling pathway, which regulates appetite and energy homoeostasis (Table S3).

### Variants with unique genotype–phenotype correlations in the Arab world

Out of the 76 captured variants that have been reported to be significantly associated with obesity in Arab world countries, 19 variants were found to exhibit genotype-phenotype correlations that are unique to Arabs, given the fact that they were reported to be distinctively associated with obesity among Arab, but not non-Arab populations. Notably, these variants have been associated with other clinical conditions (i.e., different from obesity), in non-Arab populations.

The variants with unique genotype–phenotype correlations in Arabs were primarily located in *LEP* and *IL-6*, with two variants in each of the two genes. The two *LEP* variants rs7799039 and rs11761556, which have been associated with obesity in Tunisia (Table 1), were reported to be associated with breast cancer and systemic lupus erythematosus in Chinese and Mexican populations (Table 1). The two *IL6* variants, rs1800795 and rs1554606, reported in Egypt and Saudi Arabia, respectively (Table 1, Table S3, Fig. 3), are considered unique to the Arab region according to genotype–phenotype correlation, as they were only reported in other ethnic populations with inflammatory conditions, such as osteoporosis in Taiwan [53] (Table 1, Table S3). The *IL6* gene encodes interleukin-6 (IL-6), an immune modulator and proinflammatory cytokine involved in regulation of the acute phase response [54]. *IL6* levels increase with increasing body fat content, and it plays an important role in glucose disposal, lipolysis, and fat oxidation. Therefore, impaired *IL6* represents a risk factor for increased body weight (Table S3).

Other variants that were found to be associated with obesity only in Arab countries include *MTFHD1* rs2236225, *EXT*: rs3740878, *PRDM16* rs2651899, and *TP53* rs1042522 (Table 1, Table S3, and Fig. 3). These variants have been significantly associated with congenital heart defects in Iran, type 2 diabetes mellitus in China, migraine in China, and cervical cancer in China [55–58] (Table S3). This is not surprising because obesity is known to progress or cause other diseases. Therefore, it is not unexpected to identify common genes and mechanisms between obesity and other associated diseases.

Notably, *PRDM16:* rs2651899 variant that conferred the strongest association with obesity (cOR = 44.60, 95% CI: 11.60–172.02, *P* = 0.0001) (Table S3), has not been associated with obesity in any non-Arab ethnic population. It has been primarily associated with a higher risk of migraines, as revealed by a large GWAS of over 5000 patients [59].

Finally, we investigated the significant and unique genes and variants associated with obesity in Arab countries and further classified them as common or unique in terms of the manifested phenotype among other ethnic groups. Nevertheless, further studies need to be performed to highlight the significance of these genes in contributing to the direct pathogenesis of obesity. This review may help build a platform for designing a gene panel to test the susceptibility of Arab patients to obesity and will also be useful globally for molecular diagnostic purposes.

### Variants shared with other ethnic groups in terms of genotype–phenotype correlation

Out of the 76 captured variants that have been reported to be significantly associated with obesity in Arab countries worldwide, 55 variants located in 35 different genes were found to exhibit genotype-phenotype correlations that are shared between Arab and non-Arab populations, given the fact that they were reported in association with obesity in both Arabs and non-Arabs.

Most of the variants in this category were located in *ADIPOQ*, the second most common gene according to the number of obesity-associated variants among Arabs. *ADIPOQ* had seven variants, all of which were found to be shared with other ethnic populations according to genotype–phenotype correlation (Table S3). These seven variants were reported in Tunisia, Egypt, and Jordan (Table 1, Fig. 3). Among them, is the intron variant rs1501299, captured in obese patients from Egypt, Jordan, and Tunisia (Table 1). This variant was previously reported to be among the top-ten obesity risk-associated SNPs in a randomly recruited cohort of Spanish children and adolescents aged 5–17-years-old [60]. *ADIPOQ* gene is a critical gene that contributes to obesity and has been investigated worldwide, having been reported in other ethnic groups, such as Japanese and Portuguese populations in association with obesity [61, 62], as well as being reportedly associated with both type 2 diabetes mellitus and obesity in South India [63] (Table S3).

The remaining variants in this category were primarily located in *FTO* and *LEP*, with five and four variants in each gene, respectively. *FTO* is the third most common gene according to the number of obesity-associated identified variants in Arabs (Table 1, Table S3). *FTO* variants (rs11642841, rs1421085, rs17817449, rs3751812, rs9939609), which have been linked to obesity in six Arab countries (Table 1, Fig. 3), and according to the clinical phenotype, they are considered as “common variants”; as they were found to be associated with obesity in other non-Arab countries, including China, Croatia, and Brazil (Table S3). *LEP* variants (c.313 C > T, rs1349419, rs2167270, (TTTC) *n* polymorphism) have been associated with obesity in other non-Arab ethnic countries, including Pakistan, Mexico, Malaysia, and India [36, 64–66] (Table S3).

### Strengths and limitations

The strengths of this study lie in the fact that it is the first systematic study to comprehensively summarize all reported obesity-associated genetic polymorphisms in the Arab world. We conducted a comprehensive search using stringent predetermined inclusion and exclusion criteria and thorough analyses of each article included. Despite precluding a meta-analysis, key findings were reported narratively. Nevertheless, our study has some limitations. First, it is noteworthy to mention that obesity is a complex multifactorial disease that can progress or cause other diseases, such as type 2 diabetes mellitus hypertension, dyslipidemia, cardiovascular disease, and nonalcoholic fatty liver disease [67, 68]. Therefore, most studies in the field of obesity include other metabolic syndromes, and most of the time, the patient group had obesity as well as other metabolic syndromes. This makes investigating the genetic polymorphisms that are only linked to obesity more challenging; thus, many of the significantly reported genes will also be associated with other metabolic syndromes. Moreover, because of recently available genome-wide association studies, over 227 genetic variants have been associated with polygenic obesity. However, the available studies are often heterogeneous with regard to their design and operational quality, which adds to the complexity of evidence and conclusion synthesis. Furthermore, the included studies also varied vastly in sample size and variables measured, as well as the degree to which they controlled for confounders. In addition, despite thoroughly searching the existing literature on obesity-associated genetic polymorphisms in the Arab world, we found relatively few genetic association studies on obesity risk in the Arab world.

## Conclusions

The epidemic of obesity has been largely attributed to changes in lifestyle habits established over the past three decades. These changes are mainly attributed to dietary and behavioral trends such as decline in physical activity. However, the obesogenic environment is not sufficient to determine the presence of obesity, a combination with genetic predisposition is required. This systematic review was designed to comprehensively assess all genetic variations significantly associated with obesity risk in Arab countries. Our findings indicate that Arabs have distinct disease susceptibility genotypes that are responsible for obesity. Although some of the obesity-associated variants mentioned in this study have been reported in other ethnic groups, the complex gene-environment interactions allow for enrichment of these genotypes, thus predisposing individuals of these ethnic groups to obesity. Our study creates a paradigm for future well-controlled epidemiological studies that will allow dissection of the genetic architecture that renders Arabs susceptible to obesity and thus may in the future serve as a platform to design a gene panel for early, accurate, and presymptomatic diagnosis of obesity. Despite our comprehensive search strategy, the dearth of genetic association studies related to obesity in the Arab world suggests a need for additional well-designed genetic association studies to serve as the basis for understanding the genetic architecture that renders Arab populations susceptible to obesity.

## Supplementary information

Table S1

Table S2

Table S3

Table S4
