# Supplementary material for: Genetic polymorphisms associated with obesity in the Arab world: a systematic review
Source: Int J Obes (Lond). 2021 Jun 15;45(9):1899–913. doi: 10.1038/s41366-021-00867-6 (PMC8380539; doi:10.1038/s41366-021-00867-6)
Supplement: Supplementary file 2 — Table S2 [file 41366_2021_867_MOESM2_ESM.docx]

**Table S2**

| **Database** | **Search Strategy** |
| --- | --- |
| **PubMed** | (Obesity OR Overweight OR BMI) AND (Gene OR Genetic OR Mutation OR Variant OR SNP OR polymorphism) AND (algeria[mesh] OR egypt[mesh] OR libya[mesh] OR morocco[mesh] OR tunisia[mesh] OR south sudan[mesh] OR sudan[mesh] OR middle east[mesh:noexp] OR bahrain[mesh] OR iraq[mesh] OR jordan[mesh] OR kuwait[mesh] OR lebanon[mesh] OR oman[mesh] OR qatar[mesh] OR saudi arabia[mesh] OR syria[mesh] OR united arab emirates[mesh] OR yemen[mesh] OR algeria*[tw] OR somalia[mesh] OR mauritania[mesh] OR djibouti[mesh] OR arabs[mesh] OR comoros[mesh] OR bahrain*[tw] OR egypt*[tw] OR iraq*[tw] OR jordan*[tw] OR kuwait*[tw] OR lebanon[tw] OR lebanese[tw] OR libanaise[tw] OR yemen*[tw] OR UAE[tw] OR emirat*[tw] OR abu-dhabi[tw] OR dubai[tw] OR libya*[tw] OR morocco[tw] OR moroccan*[tw] OR oman*[tw] OR muscat[tw] OR palestin*[tw] OR gaza[tw] OR west-bank[tw] OR qatar*[tw] OR saudi*[tw] OR KSA[tw] OR syria*[tw] OR tunis*[tw] OR comoros*[tw] OR sudan*[tw] OR arabs[tw] OR arab[tw] OR arabia[tw] OR somali*[tw] OR mauritania*[tw] OR djibouti*[tw] OR levant[tw]) |
| **Scopus** | TITLE-ABS-KEY((Obesity OR Overweight OR BMI) AND (Gene OR Genetic OR Mutation OR Variant OR SNP OR polymorphism) AND (lebanon OR lebanese OR algeria* OR bahrain* OR egypt* OR iraq* OR jordan* OR kuwait* OR libanaise OR comoros* OR yemen* OR dubai OR (abu W/2 dhabi) OR UAE OR emirat* OR libya* OR Somalia OR morocco OR moroccan* OR Tunisia OR tunisian OR oman* OR muscat OR palestin* OR gaza OR qatar* OR saudi* OR KSA OR Syria* OR tunis* OR sudan* OR djibouti* OR somali* OR mauritania* OR levant OR arabs OR arab OR arabia)) |
| **Science Direct** | (Obesity OR Overweight OR BMI) AND (Gene OR Genetic OR Mutation OR Variant OR SNP OR polymorphism) AND (lebanon OR lebanese OR algeria* OR bahrain* OR egypt* OR iraq* OR jordan* OR kuwait* OR comoros* OR yemen* OR dubai OR (abu W/2 dhabi) OR UAE OR emirat* OR libya* OR Somalia OR morocco OR moroccan* OR Tunisia OR tunisian OR oman* OR muscat OR palestin* OR gaza OR qatar* OR saudi* OR KSA OR Syria* OR tunis* OR sudan* OR djibouti* OR somali* OR mauritania* OR levant OR arabs OR arab OR arabia) |
| **Google Scholar** | (Obesity OR Overweight OR BMI) AND (Gene OR Genetic OR Mutation OR Variant OR SNP OR polymorphism) AND (lebanon OR lebanese OR algeria* OR bahrain* OR egypt* OR iraq* OR jordan* OR kuwait* OR comoros* OR yemen* OR dubai OR (abu W/2 dhabi) OR UAE OR emirat* OR libya* OR Somalia OR morocco OR moroccan* OR Tunisia OR tunisian OR oman* OR muscat OR palestin* OR gaza OR qatar* OR saudi* OR KSA OR Syria* OR tunis* OR sudan* OR djibouti* OR somali* OR mauritania* OR levant OR arabs OR arab OR arabia) |
